# Measles

**Published:** 1905-11-25

**Authors:** 


### Hospital Clinics.

### MEASLES.

A statistical investigation of wide scope and no little merit has been carried out by Dr. George N. Wilson [1] with the object of eliciting the laws governing the appearance of measles in epidemic form, its varying severity, its mortality, and certain other subsidiary matters. Measles, as the author points out, is the most universal of zymotic fevers in this country, and leads to sufficient deaths to claim for itself a high place in the consideration of hygienists; in Aberdeen, for instance, measles has caused within the last ten years more deaths than any other single zymotic fever, and, if whooping-cough be excluded, more than all the other zymotic fevers put together.

The author is favourably placed in the matter of this investigation, for he has had access to the data accumulated in Aberdeen during a period of compulsory notification of the disease. In 1881 the Corporation of Aberdeen obtained powers to compel the notification of infectious diseases, including measles and whooping-cough; and this was carried out for ten years, the notification being made by the medical attendant only. At this stage, and for the next ten years, dual notification (by both the medical practitioner and the householder, that is to say) was enforced by the authorities with a view to greater accuracy in the returns. This system of notification was suspended in 1903, the benefits accruing from it being considered disproportionately small in view of the cost. There has thus been a continuous notification of measles in Aberdeen for twenty consecutive years, and it is upon the data thus rendered available that Dr. Wilson has drawn for the information given in his paper. It is observed that the returns do not, of course, claim scientific accuracy; but this, after all, is not required: it is enough to know that the system of notification was rigidly carried out, prosecutions being instituted when defaulters were discovered. Under such circumstances it is fair to assume that the minority of cases which escaped notification, either from a wilful or accidental cause, was for practical purposes a negligible one.

Firstly, as regards the attack-incidence of measles. In the twenty years under review there were notified in Aberdeen 40,374 cases, the sex distribution in the series being curiously even—*i.e.* males 20,287, and females 20,087. The relative frequency of the disease during the different months of this series of years is well indicated in a carefully prepared chart, which appears to show, in addition, a definite cyclical tendency in the epidemic prevalence of measles. In Aberdeen, at least, measles assumes a notably epidemic form every second year with a most uncommon regularity. For instance, the rate calculated per 100,000 of the population was never higher than 20 in 1883; in 1884 it rose rapidly to well over 1,100, falling as rapidly almost to zero in 1885. Throughout the greater part of 1885 and 1886 the rate remained very low, never above forty that is to say, but an epidemic originating towards the close of 1886 took the figure rapidly to over 1,400. A rapid fall again followed, and throughout the latter half of 1887 and the whole of 1888 the figure remained almost at zero. Two small epidemics then occurred in two successive years, and broke the accuracy of the sequence. From the middle of the year 1890 to the middle of 1892 measles was practically non-existent in Aberdeen, but in the latter year began the record epidemic of the series, in which the attack-incidence reached the monstrous total of 1,600 per 100,000 of population. The biennial periodicity seems to have been re-established by this epidemic, and continues undisturbed to the conclusion of the series of years under notice.

In the construing of this chart the author observes that "the height of the highest peak in an epidemic does not always indicate the extent or mass of it. Some epidemics rise quickly to a great height and fall as quickly, while others which do not rise so high cover a longer period of time—*e.g.* the epidemic of 1892 attained the giant height of 1,613 per 100,000 of population in three months, and fell almost to zero in four months, thus covering a period of seven months, whereas the epidemic of 1900 rose to 694 per 100,000 of population in two months, but took eleven months to fall to zero. The epidemic of 1900, as a matter of fact, represented more cases than that of 1892."

The chart suggests also a periodicity among the epidemic years themselves, a specially high epidemic showing itself every sixth year, that is to say at every third epidemic. It seems besides, as is indeed to be expected, that the longer the interval of low prevalence of the disease, the greater the height likely to be attained by the next succeeding epidemic wave.

A second chart shows the seasonal variation in the attack incidence of measles. This is distinctly a seasonal disease and reaches its point of greatest prevalence during the colder months of the year. The chart is constructed to show the average monthly incidence of measles throughout the twenty years with which the paper deals, and indicates, besides the general cold-weather rise, that there are two maxima, one—the higher—in December, and the other in March. A long drop occurs in April, from which month a regular step-like descent of the record leads to the well-marked minimum of August, when the cases are less than one-tenth the number for December. Attention is drawn in this connection to a similar chart prepared by Dr. James S. Laing, illustrating the seasonal variations of whooping-cough. The result of this investigation showed that in the case of the latter disease the month of maximum prevalence was April, that of minimum prevalence September, that the maximum was only about three times the minimum, and that the ascent from minimum to maximum was slow.

Statistics dealing with the age-incidence of measles show the results indicated by the following table. The figures represent the rate of incidence per 1,000 living at each age.

| | |
|---|---|
| First year ... ... ... ... ... | 43.7 |
| Second year ... ... ... ... ... | 85.6 |
| Third year ... ... ... ... ... | 85.6 |
| Fourth year ... ... ... ... ... | 79.3 |
| Fifth year ... ... ... ... ... | 75.4 |
| Sixth year ... ... ... ... ... | 86.3 |
| Seventh year ... ... ... ... ... | 75.2 |
| Eighth year ... ... ... ... ... | 47.3 |
| Ninth year ... ... ... ... ... | 21 |

From the ninth year onward there ensues a rapid drop in the incidence of measles, due no doubt to a large extent to the protection afforded by a previous attack. The chief practical conclusion to be drawn from this study of age-incidence is "that the great bulk of children have before the completion of their eighth year taken measles, at any rate in a town like Aberdeen, and it is needless in a threatened epidemic to close schools in so far as children above eight years are concerned."

As regards the distribution of measles between the sexes, it appears that there is but little difference, but such as it is, it favours the female rather than the male. Liability to second attacks increases for some years as age advances; thus the proportion of second attacks per 1,000 cases notified is as follows:—

| | |
|---|---|
| From birth to the fifth year ... ... ... | 9.6 |
| From the fifth to the fifteenth year ... ... | 43.8 |
| From the fifteenth to the twenty-fifth year ... | 98.0 |

Also, in general terms females are more prone to second attacks than males.

Epidemics of measles and whooping-cough so frequently follow each other that it has been generally supposed that the former predisposes to the latter. In the opinion of the author the relation, if any, is reversed, the declining wave of whooping-cough being more often overlapped by the rising epidemic line of measles than the opposite. Both diseases, however, tend to be biennial in their epidemic periodicity, and alternate with great regularity.

Considerable space is devoted by the author to the statistics of the mortality of measles. It will be clear upon a moment's reflection how great is the inevitable error in such a statistical compilation as is based upon the returns of the Registrar-General. Measles is a disease whose fatality is, one might almost say, incidental. The disease itself kills by the virulence of its infection in a very small proportion of cases indeed. In order properly to appreciate the destructive nature of the disease when operating in surroundings unfavourable to health, one must have experienced the frequency with which it is said at hospitals for children that the patient "has never been the same since measles," perhaps six months before. Many deaths notified, and properly notified, as tuberculosis are in reality the fruit of measles; and the same may be said of broncho-pneumonia. We incline, therefore, to regret the labour which the author has devoted to the collection of statistics so dubiously founded as these, in the nature of things, are bound to be. The unreliable nature of the data seems to be illustrated by the "very irregular height of the case-mortality," to which the author himself directs attention. Returns which place the case mortality of the same disease in the same town at figures varying from 0 to 25 per cent. are surely passing the limits of variation in the virulence of epidemic diseases. But although statistics as to the fatality of measles which are based upon the returns supplied by many hands are in all probability most incomplete, one department of the author's investigation emphasises the hygienic deficiencies of crowded tenements. He has drawn up a chart in which the case-mortality per cent. of cases notified is given for tenements of varying sizes. The result is as follows: In tenements consisting of one room, whose average number of inmates is 4.1, deaths = 6.8 per cent. of cases notified; in tenements consisting of two rooms, whose average number of inmates is 5.2, deaths = 3.0 per cent. of cases notified; in tenements consisting of three rooms, whose average number of inmates is 5.8, deaths = 1.8 per cent. of cases notified; in tenements consisting of four rooms, whose average number of inmates is 6.2, deaths = .9 per cent. of cases notified.

[1] Public Health, November, 1905.

